# Supplementary material for: Systematic identification of regions where DNA methylation is correlated with transcription refines regulatory logic in normal and tumour tissues
Source: Nucleic Acids Res. 2025 Sep 30;53(18):gkaf949. doi: 10.1093/nar/gkaf949 (PMC12481017; doi:10.1093/nar/gkaf949)
Supplement: gkaf949_Supplemental_Files [file gkaf949_supplemental_files.zip › supplementary_figure_legends_with_figures.pdf]

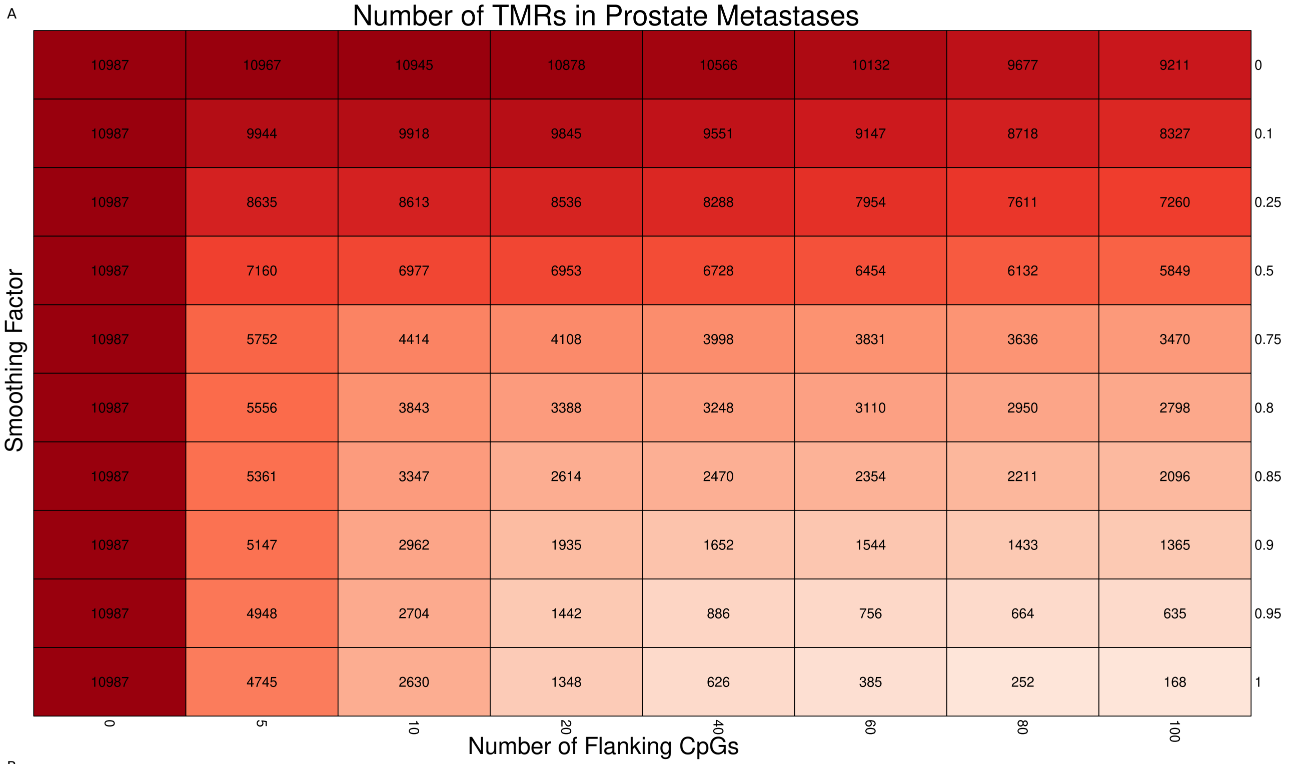

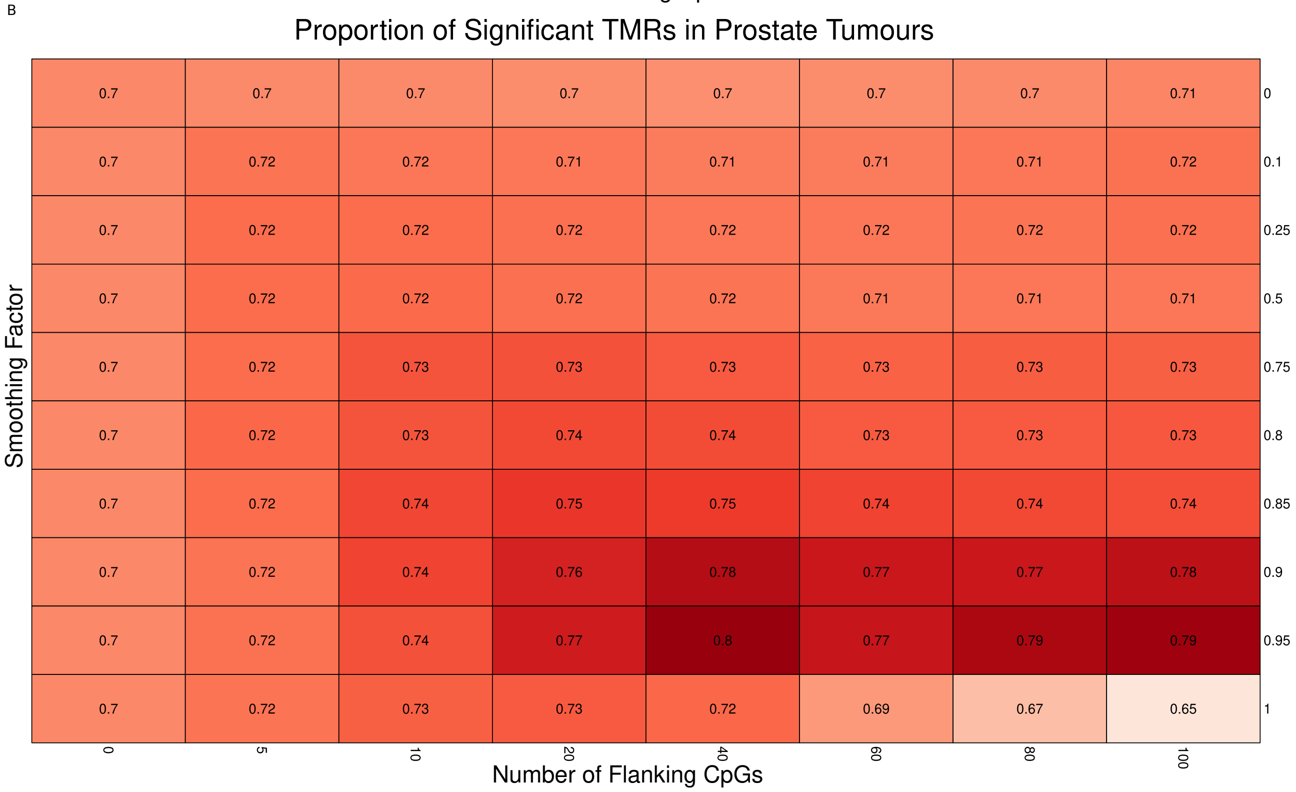

Supplementary Figure 1: (A) The number of TMRs found in prostate metastases using different combinations of the number of flanking CpGs used to construct windows and the smoothing factor used for the exponential moving average. (B) The proportion of TMRs identified in prostate metastases using different parameter combinations resulting in significant correlations when evaluating their correlations in prostate tumour samples. 10 flanking CpGs and a smoothing factor of 0.75 seemed to give a good trade-off between the number of TMRs discovered and the proportion of these that gave significant correlations.

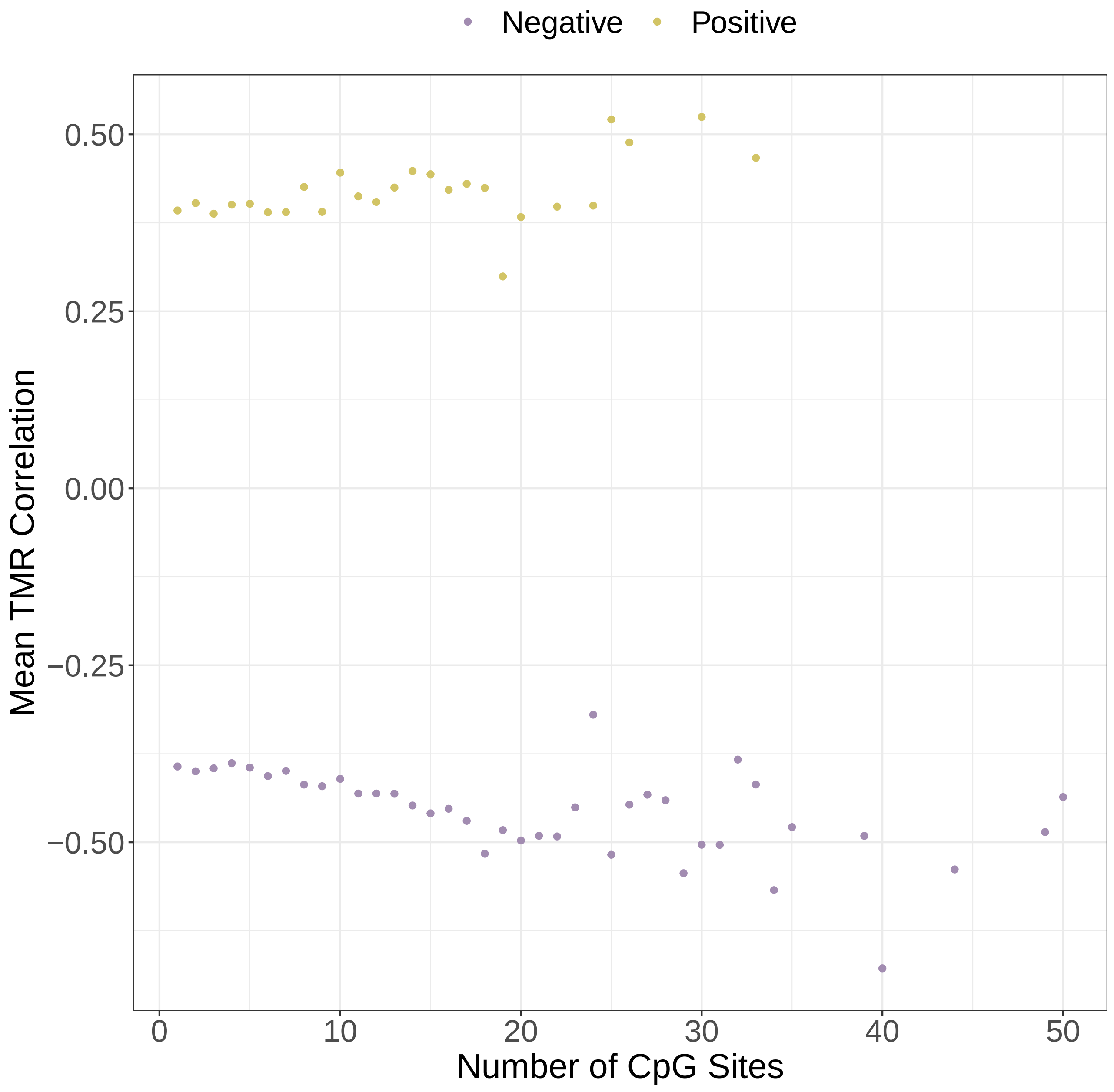

Supplementary Figure 2: The mean methylation-transcription correlations for TMRs identified in normal prostate samples grouped by the number of CpG sites that they overlap. There was a Spearman correlation value of 0.3 (p-value < 2.2e-16) between the number of CpG sites and the absolute methylation-transcription correlations of TMRs.

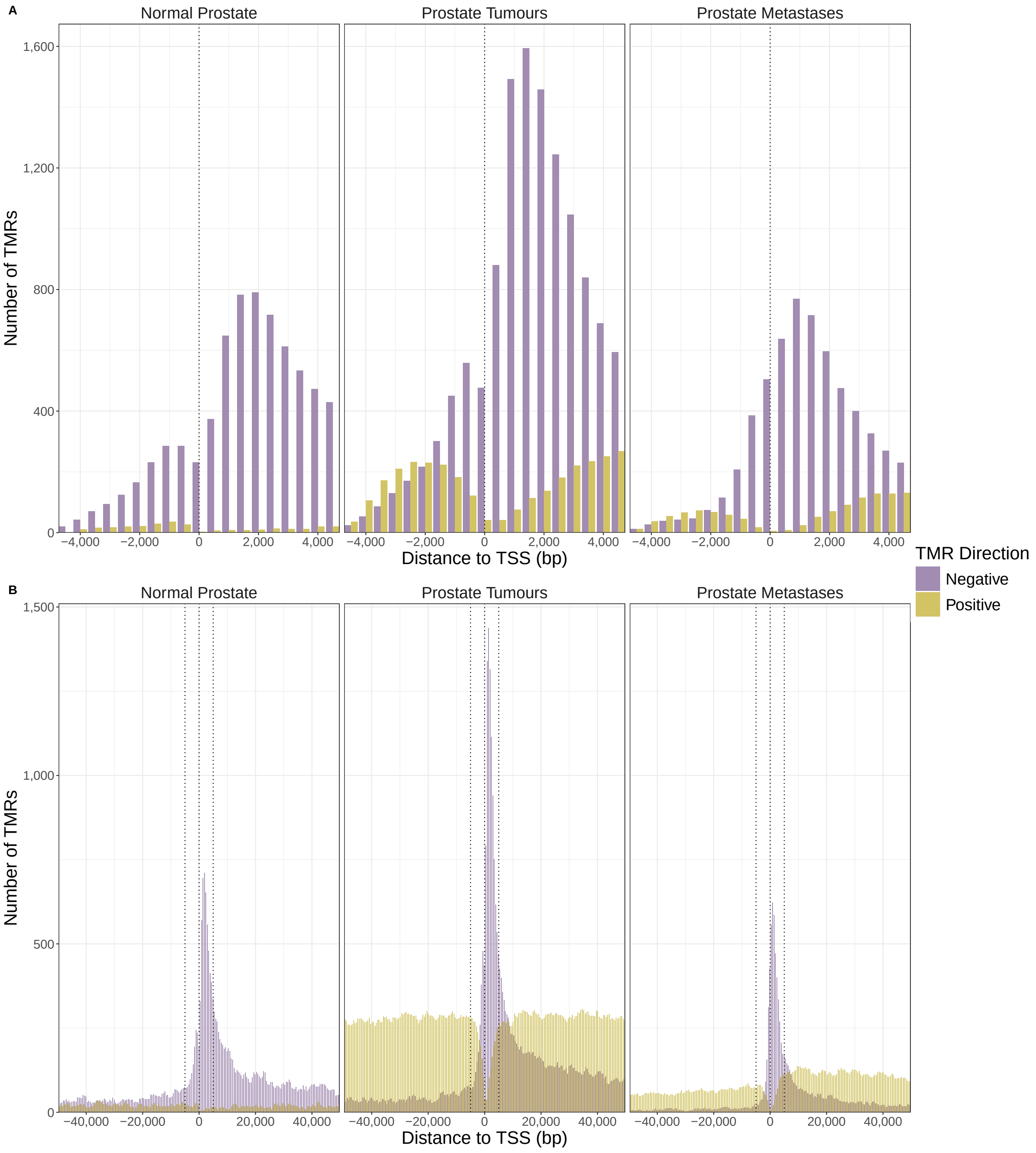

Supplementary Figure 3: The relative location of negative and positive TMRs in normal prostate, prostate tumours and prostate metastases within +/- 5 KB (A) and +/- 50 KB (B)of the TSS without removal of TMRs in poorly mappable regions The regions around TSS were divided into 500 bp bins and the number of times TMRs overlapped these bins were counted. The x-axis shows the distance from the center of bins to the TSS. The dotted line indicates the location of the TSS. There are a huge number of positive TMRs found surrounding the TSS in prostate tumour and metastasis samples.

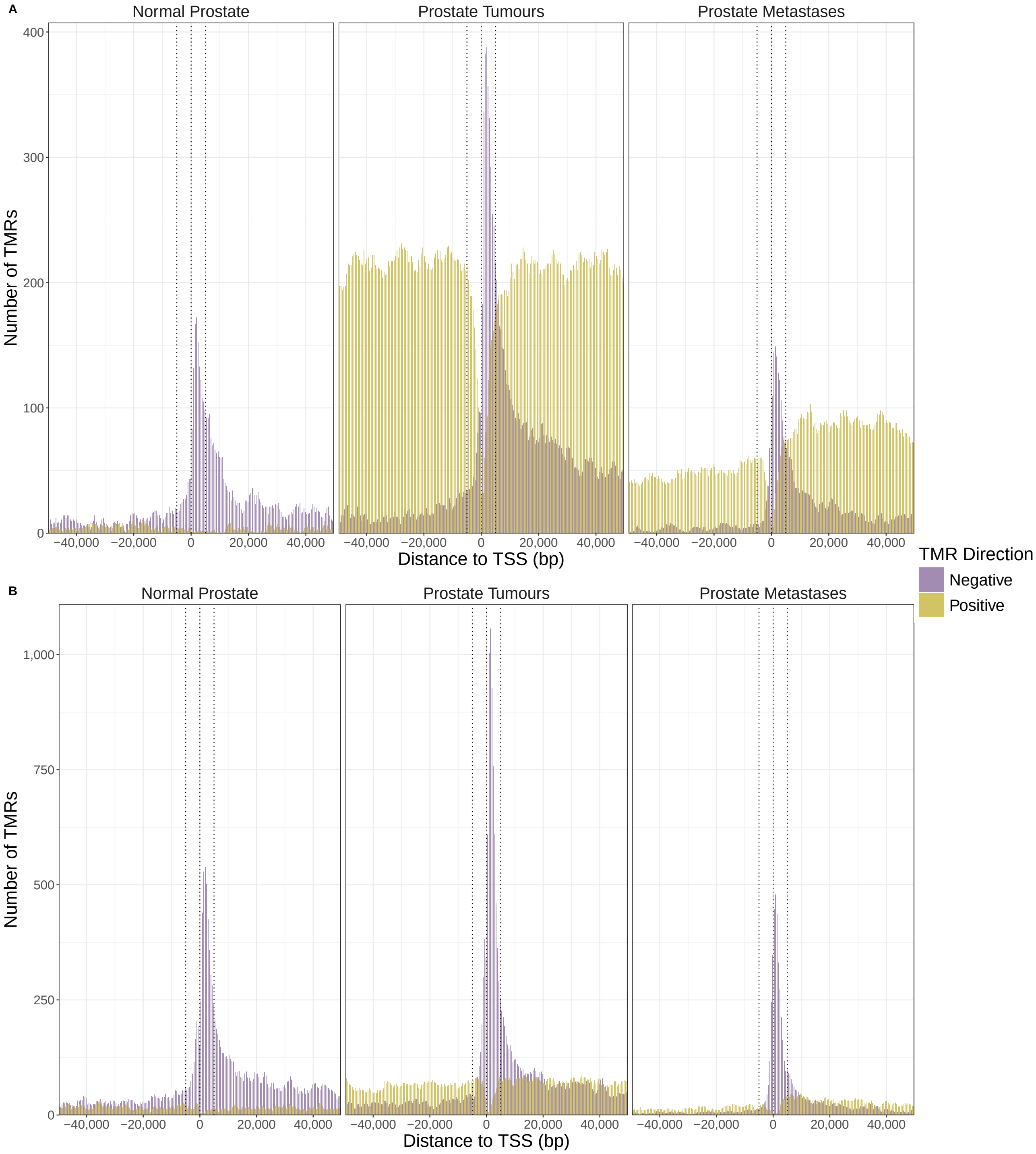

Supplementary Figure 4: (A) Distribution of TMRs which overlap poorly mappable regions within +/- 50 kb of TSS . (B) Distribution of TMRs which overlap highly mappable regions within +/- 50 kb of TSS. Axes and colours are as in Supplementary Figure 3. Dotted lines in top panels show the location of -5 kb and + 5 kb.

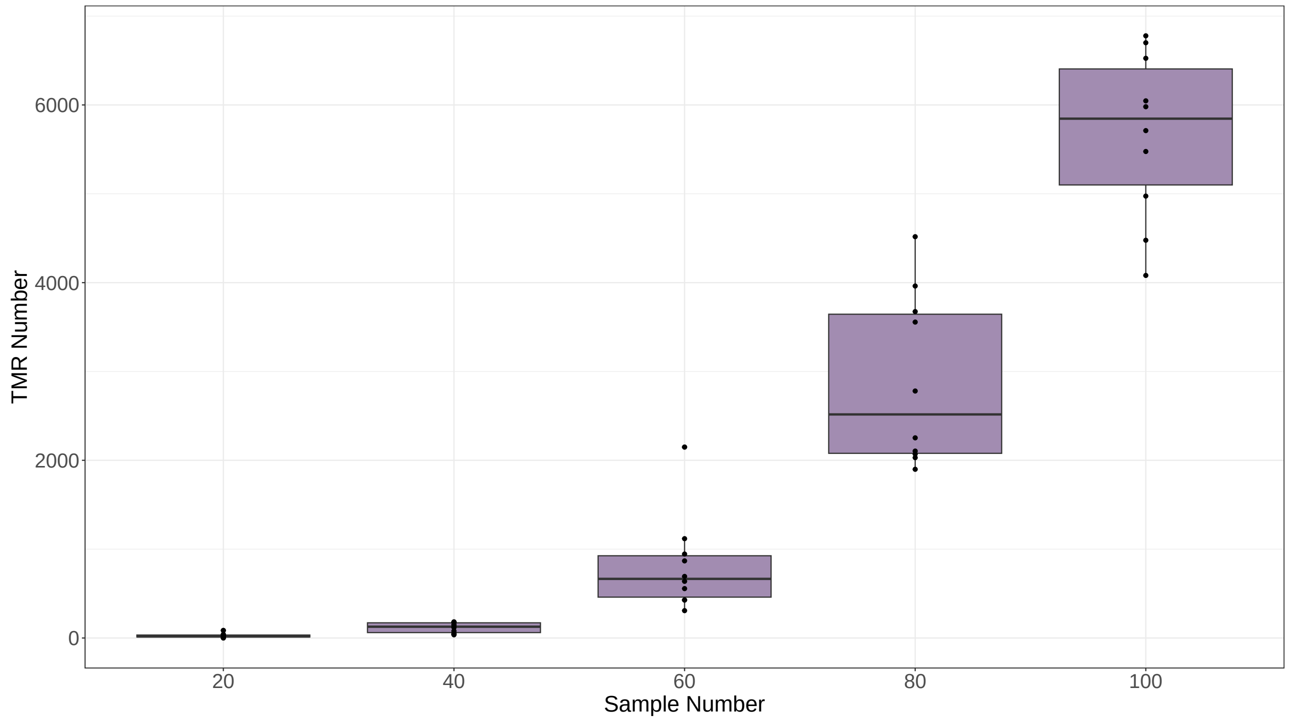

Supplementary Figure 5: The number of TMRs identified in prostate tumour samples with different sized subsets of samples. 10 random samples were used for each subset size. Below about 60 samples, the number of TMRs identified is relatively small.

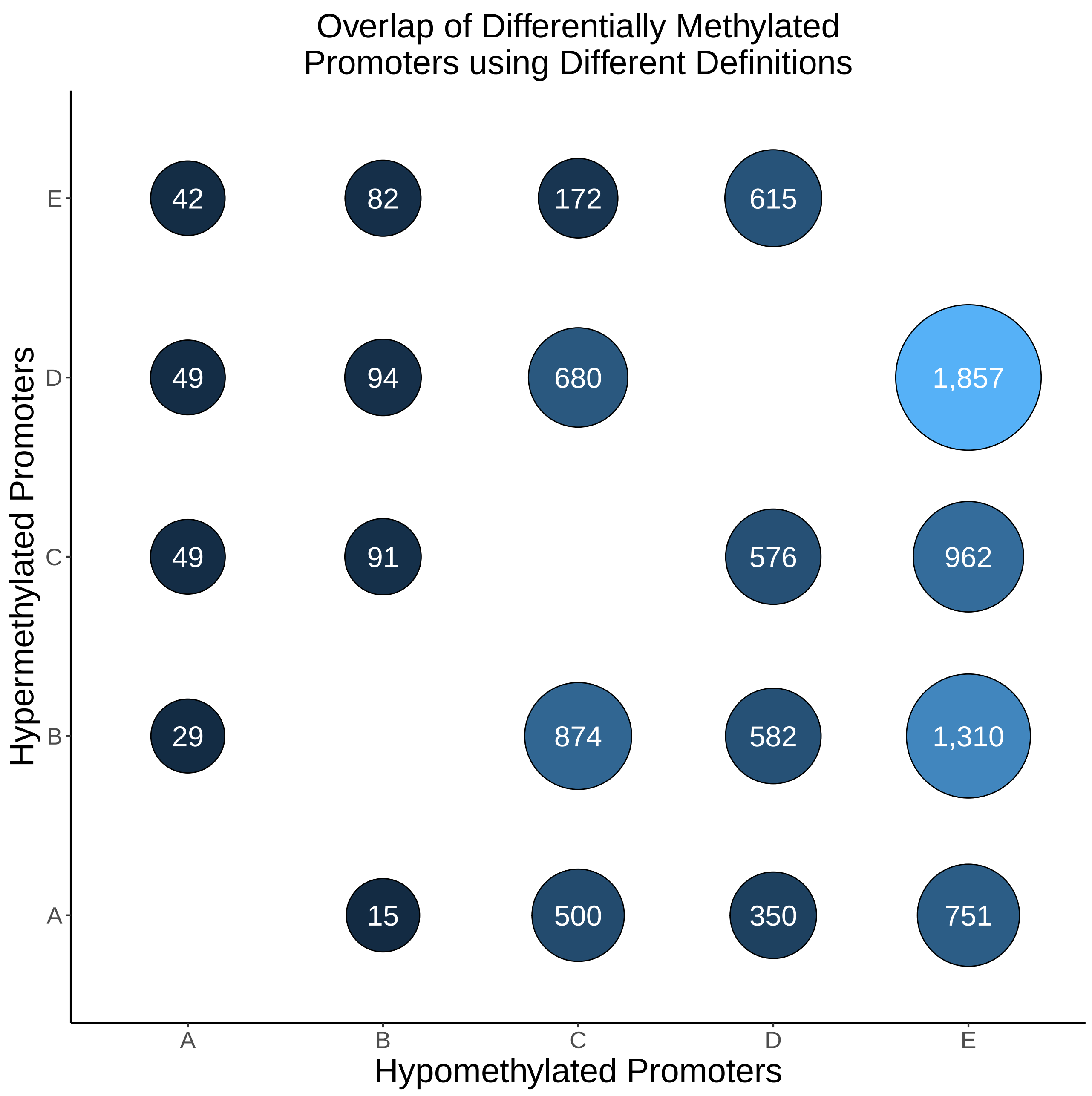

Supplementary Figure 6: The intersection sizes of hypermethylated promoters identified using one of five different promoter definitions with hypomethylated promoters identified using another one of the definitions

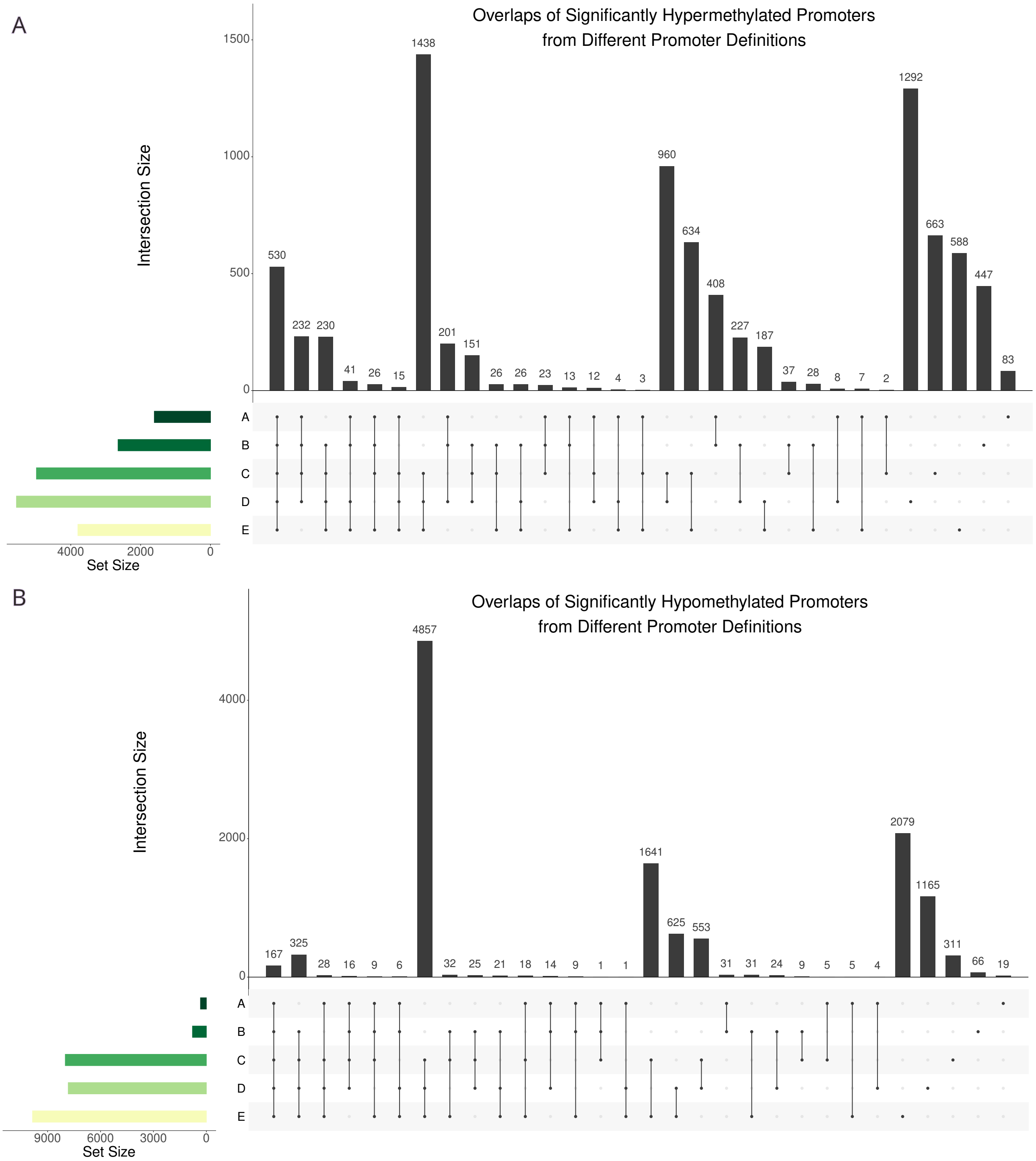

Supplementary Figure 7: UpSet plots showing the intersection of hypermethylated (A) and hypomethylated (B) promoters resulting from the use of different promoter definitions.

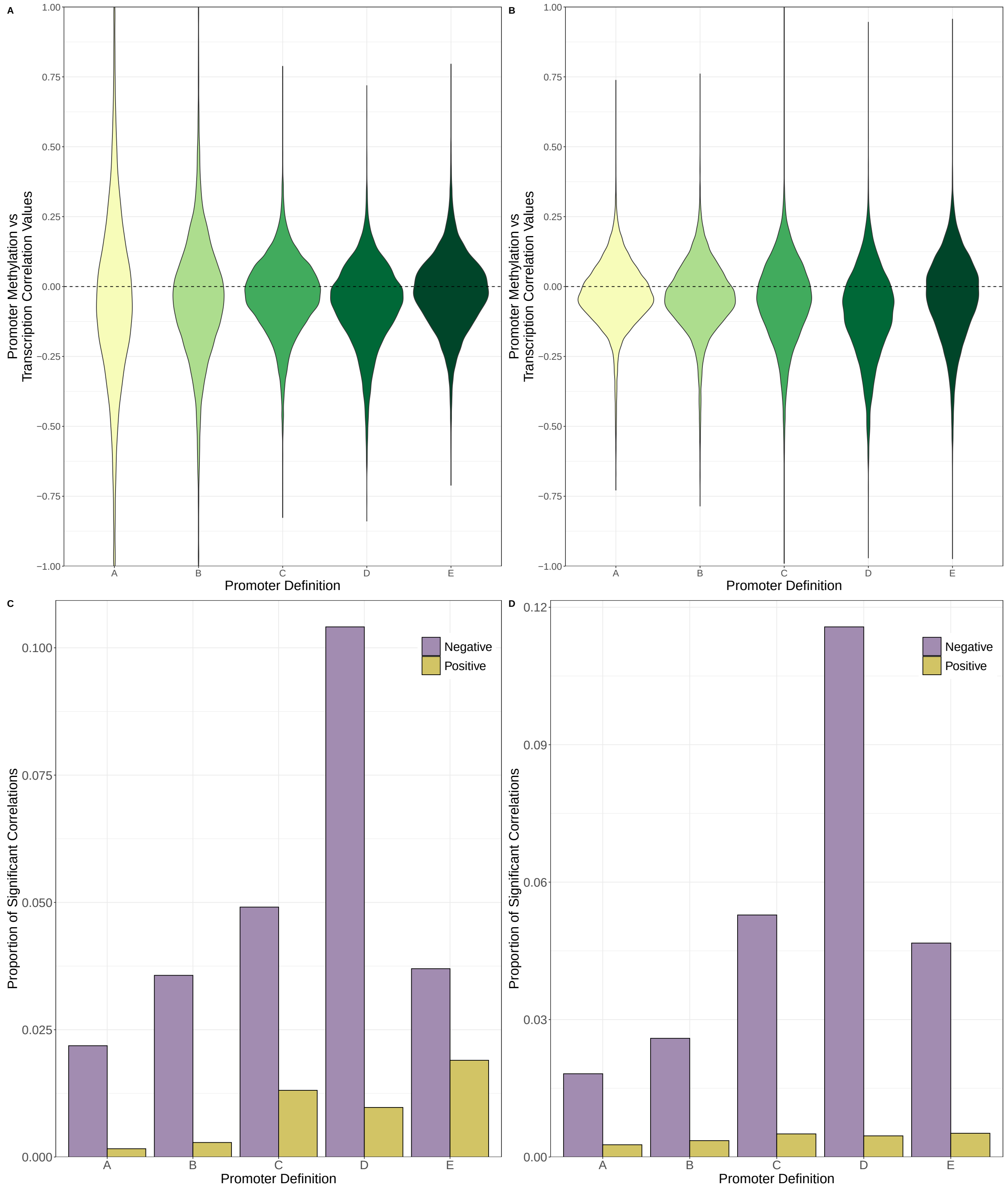

Supplementary Figure 8: The distribution of promoter methylation-transcription Spearman correlation values for all protein-coding transcripts using each of the 5 different promoter definitions in prostate tumour samples (A) and prostate metastasis samples (B). The proportion of statistically significant correlations for each promoter definition divided into negative and positive correlations in prostate tumour samples (C) and in prostate metastasis samples (D).

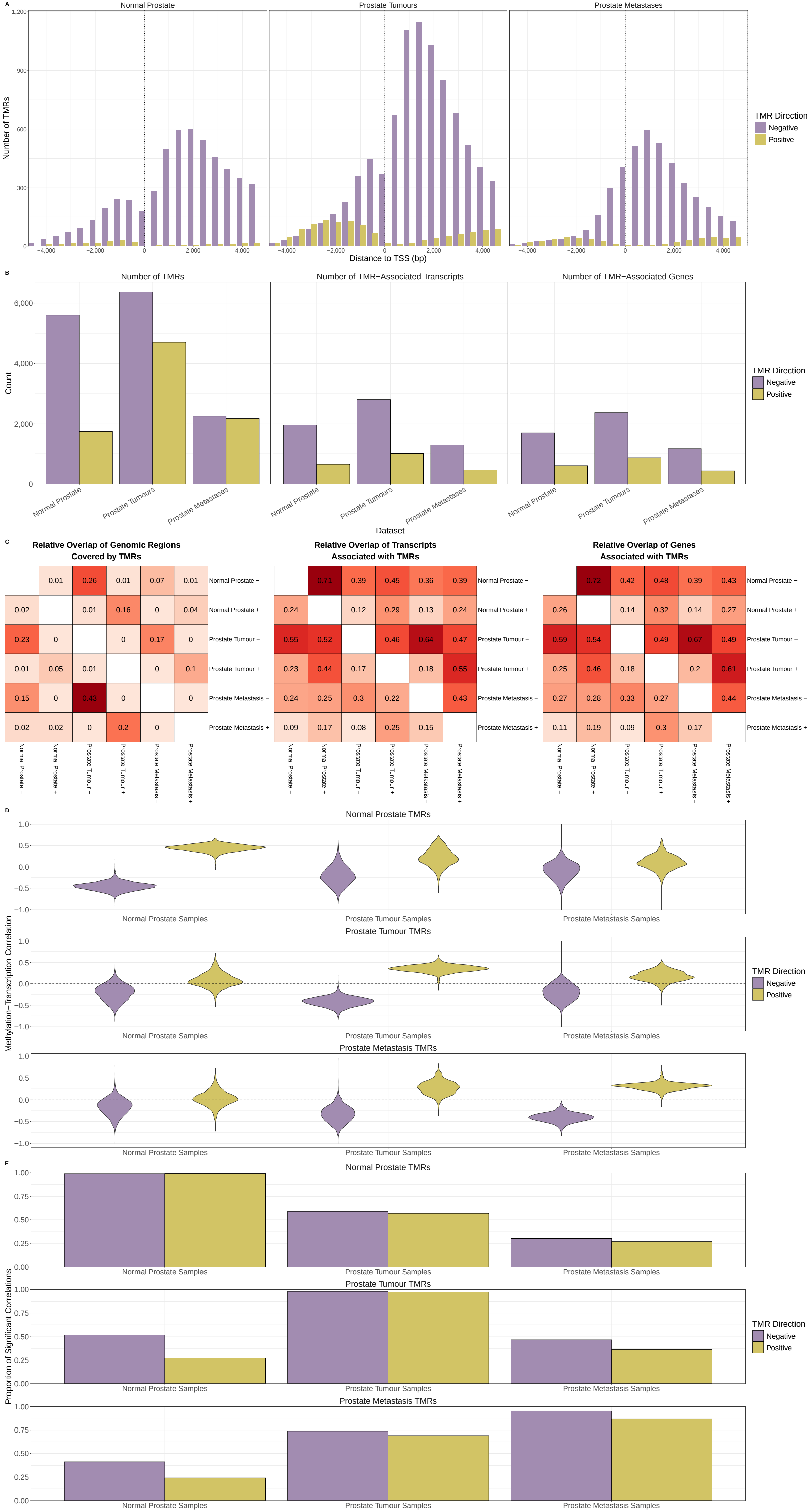

A
Normal Prostate
Prostate Tumours
Prostate Metastases
Number of TMRs
Distance to TSS (bp)
TMR Direction
Negative
Positive
B
Number of TMRs
Number of TMR−Associated Transcripts
Number of TMR−Associated Genes
Count
Normal Prostate
Prostate Tumours
Prostate Metastases
Dataset
C
Relative Overlap of Genomic Regions Covered by TMRs
Relative Overlap of Transcripts Associated with TMRs
Relative Overlap of Genes Associated with TMRs
Normal Prostate −
Normal Prostate +
Prostate Tumour −
Prostate Tumour +
Prostate Metastasis −
Prostate Metastasis +
D
Normal Prostate TMRs
Prostate Tumour TMRs
Prostate Metastasis TMRs
Methylation−Transcription Correlation
Normal Prostate Samples
Prostate Tumour Samples
Prostate Metastasis Samples
E
Proportion of Significant Correlations

Supplementary Figure 9: (A) Location of TMRs within +/- 5 kb of the TSS. Regions were divided into 500 bp bins and the number of times TMRs overlapped these bins is displayed in normal prostate samples, prostate tumour samples and prostate metastasis samples. The x-axis shows the distance from the center of bins to the TSS. The dotted lines indicate 5 kb upstream, the TSS (0) and 5 kb downstream. (B) The number of negative and positive TMRs discovered within +/- 5KB of TSS and numbers of different transcripts and genes associated with TMRs in normal prostate, prostate tumour samples and prostate metastasis samples. (C) Heatmaps of the relative overlaps of TMRs and TMR-associated transcripts and genes from normal prostate, prostate tumour samples and prostate metastasis samples. For TMR overlaps, a TMR from one group was defined as overlapping TMRs from another group if at least 25% of its sequence was located in TMRs from the other group. The relative overlap of one group with another was then defined as the proportion of TMRs from the first group (corresponding to rows) which overlapped the second (corresponding to columns). For example, 43% of the negative TMRs identified in the prostate metastasis samples overlap those identified in the prostate tumour samples. (D) Distributions of correlation values of TMRs with their associated transcript. Each panel shows the correlation values for TMRs identified in one of the datasets in each of the three datasets: the dataset in which it was identified as well as the other two datasets. For example, the central plot in the bottom panel shows the correlation values between TMR methylation and expression of the associated transcript in prostate tumour samples for negative and positive TMRs identified in prostate metastasis samples. (E) Proportion of statistically significant correlations between methylation of TMRs and their associated transcript. Like in panel D, each panel represents the correlation values for TMRs identified in one of the datasets in each of the three datasets.

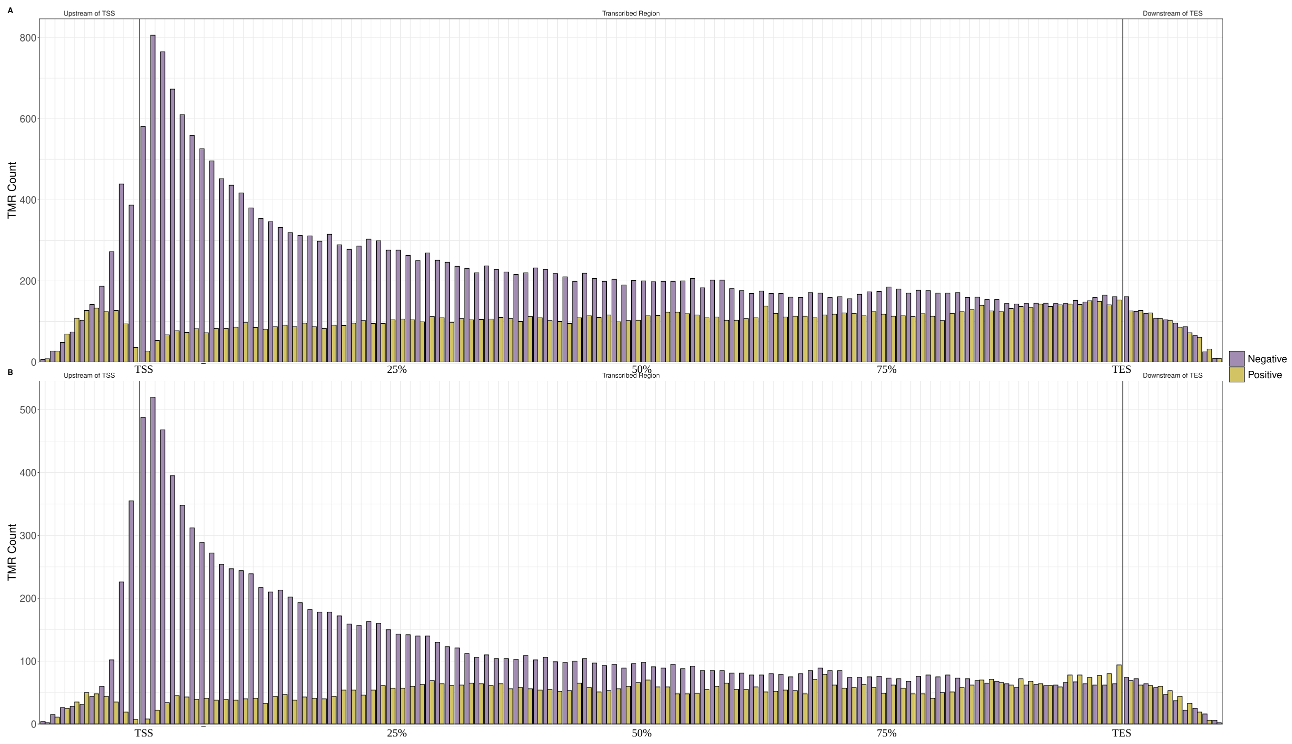

Supplementary Figure 10: (A and B) Metagene plot showing the location of TMRs identified in prostate tumour samples (A) and prostate metastasis samples (B) within transcribed regions or regions within 5 kb upstream of the TSS or 5 kb downstream of the TES. Each transcribed region was divided into 100 equally sized sections while upstream and downstream regions were divided into 500 bp bins.

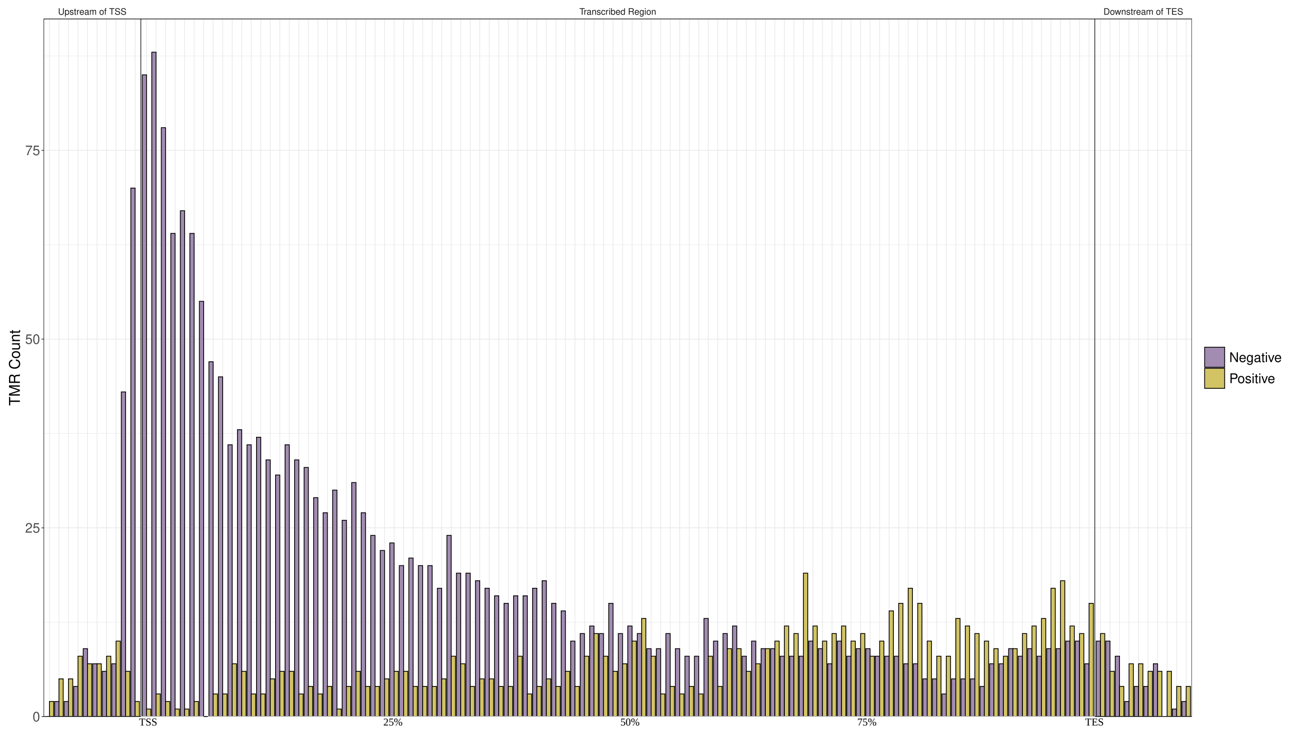

Supplementary Figure 11: (A) Metagene plot showing the location of TMRs identified intissue samples from the Roadmap Epigenomics project within transcribed regions or regions within 5 kb upstream of the TSS or 5 kb downstream of the TES Each transcribed region was divided into 100 equally sized sections while upstream and downstream regions were divided into 500 bp bins.

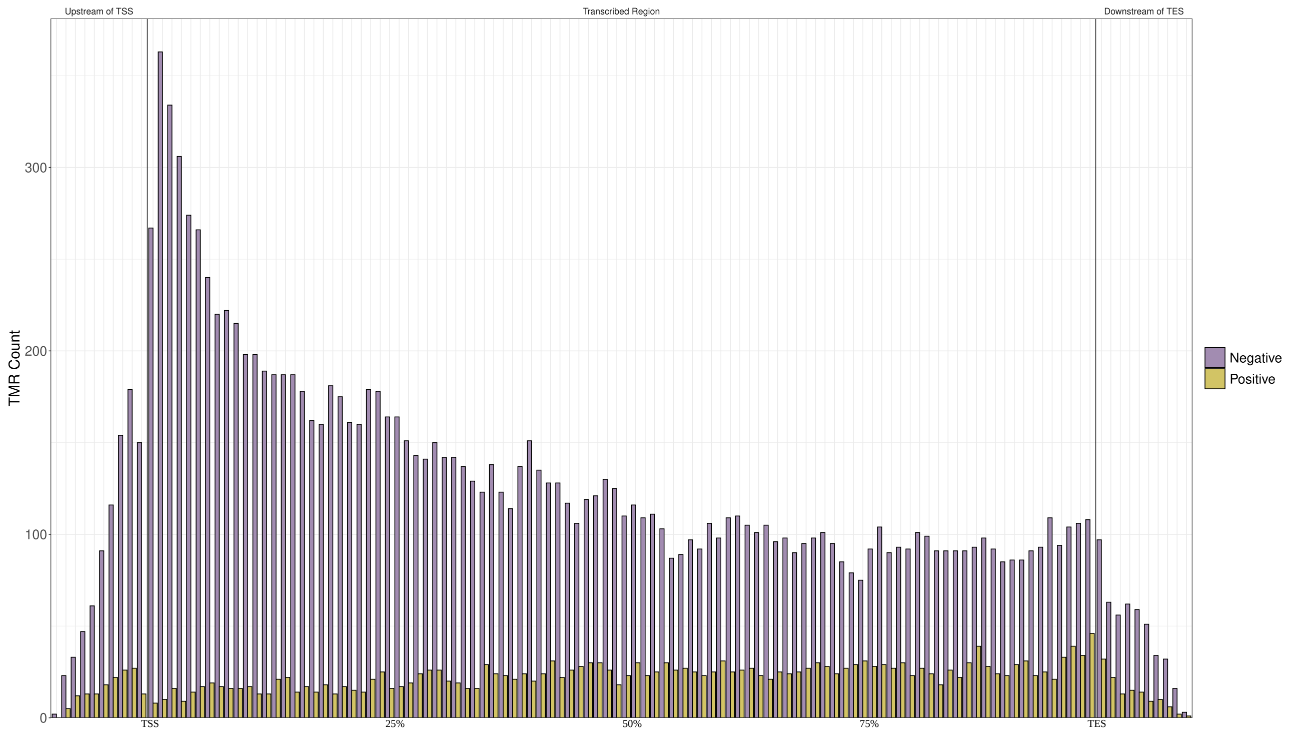

Supplementary Figure 12: Metagene plot showing the location of TMRs identified in normal prostate samples within transcribed regions or within 5 kb upstream of the TSS or 5 kb downstream of the TES for MANE transcripts. Each transcribed region was divided into 100 equally sized sections while upstream and downstream regions were divided into 500 bp bins.

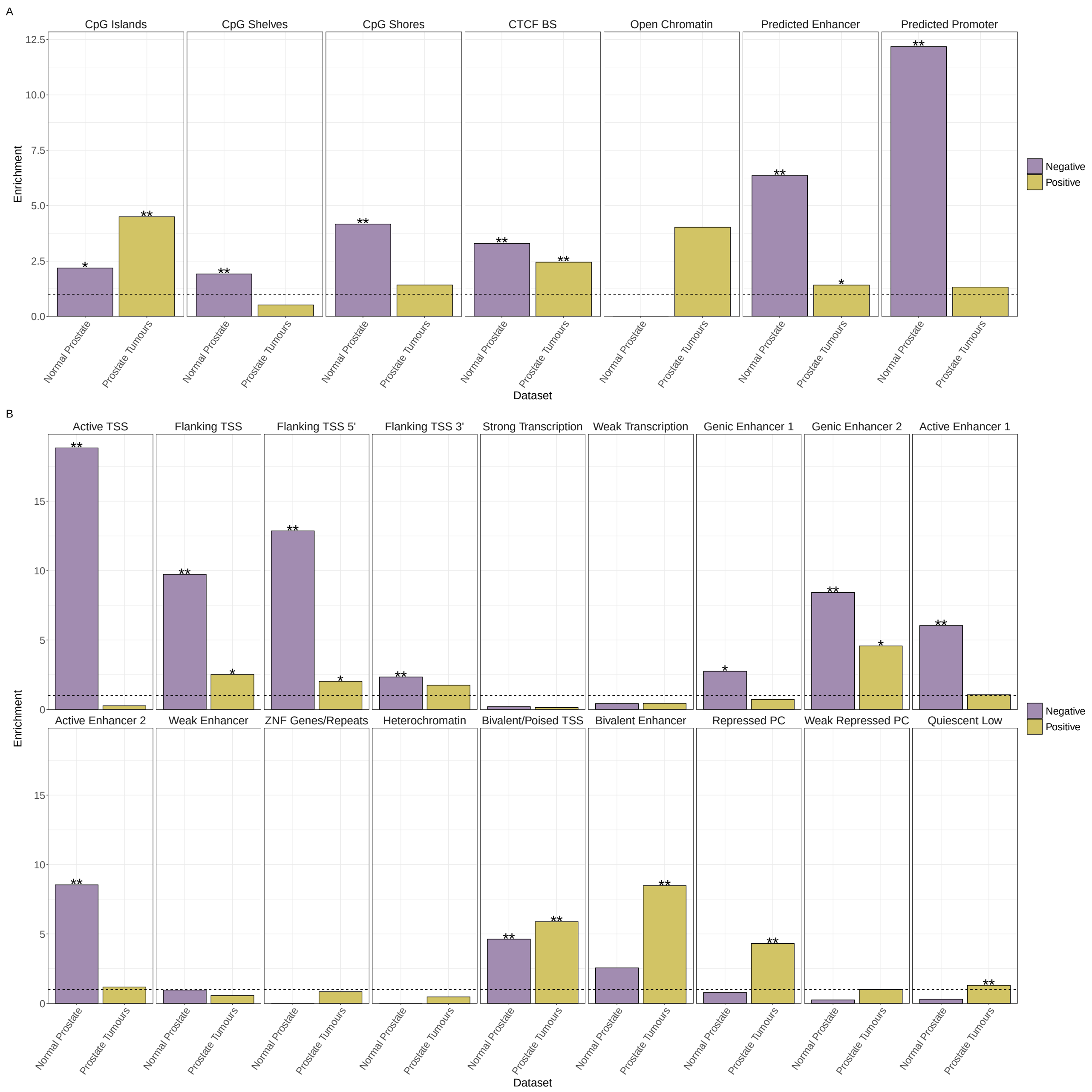

Supplementary Figure 13: (A) The enrichment of different classes of genomic regulatory elements among TMRs identified in samples from the Roadmap Epigenomics project. CTCF BS stands for CTCF binding site. (B) The enrichment of different chromatin states among TMRs identified in the samples from the Roadmap Epigenomics project, calculated similarly as with the genomic regulatory elements above.

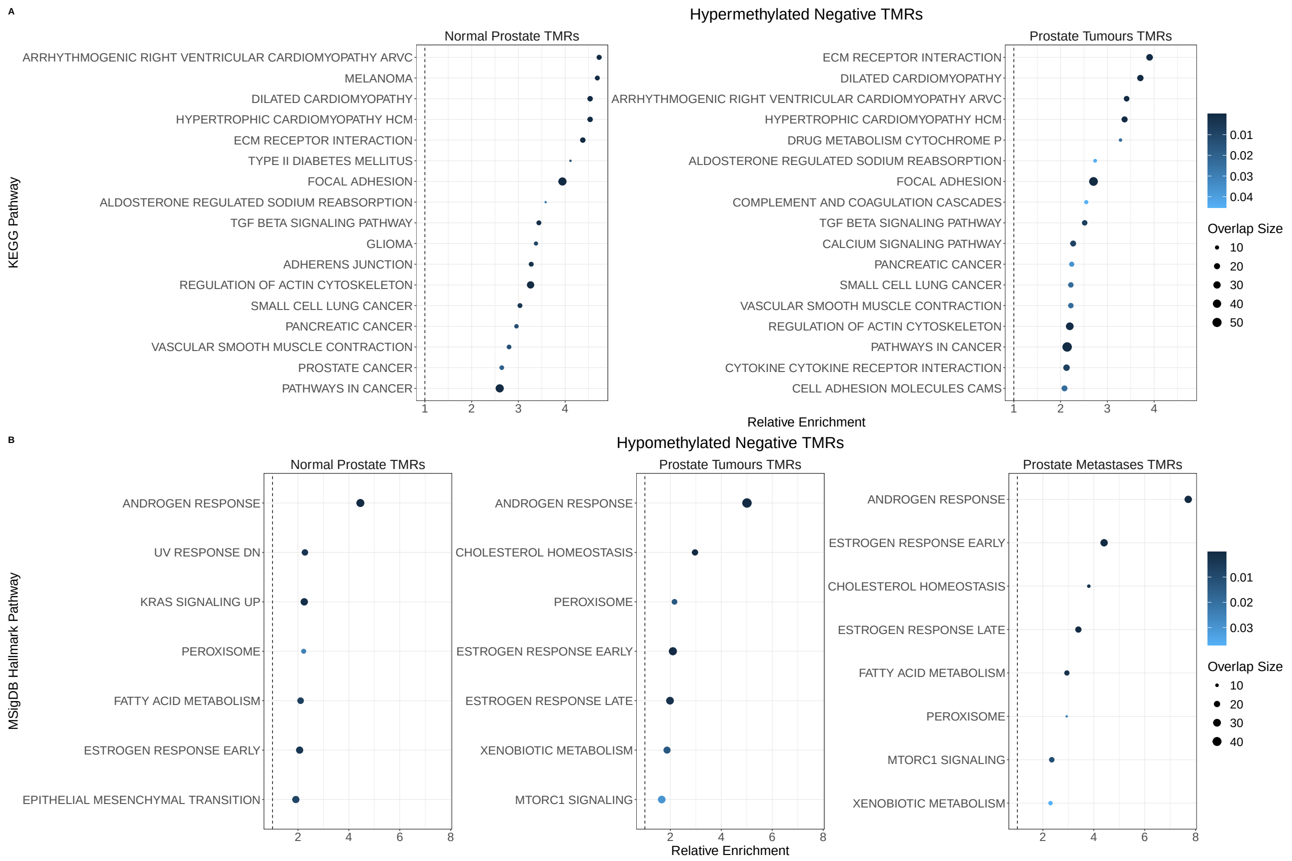

Supplementary Figure 14: (A) Overrepresentation of KEGG pathways in genes associated with hypermethylated TMRs identified in normal prostate and prostate tumour samples. (B) Overrepresentation of MSigDB Hallmark pathways in genes associated with hypomethylated TMRs identified in normal prostate, prostate tumours and prostate metastasis samples. The androgen response is the top pathway for all three datasets.

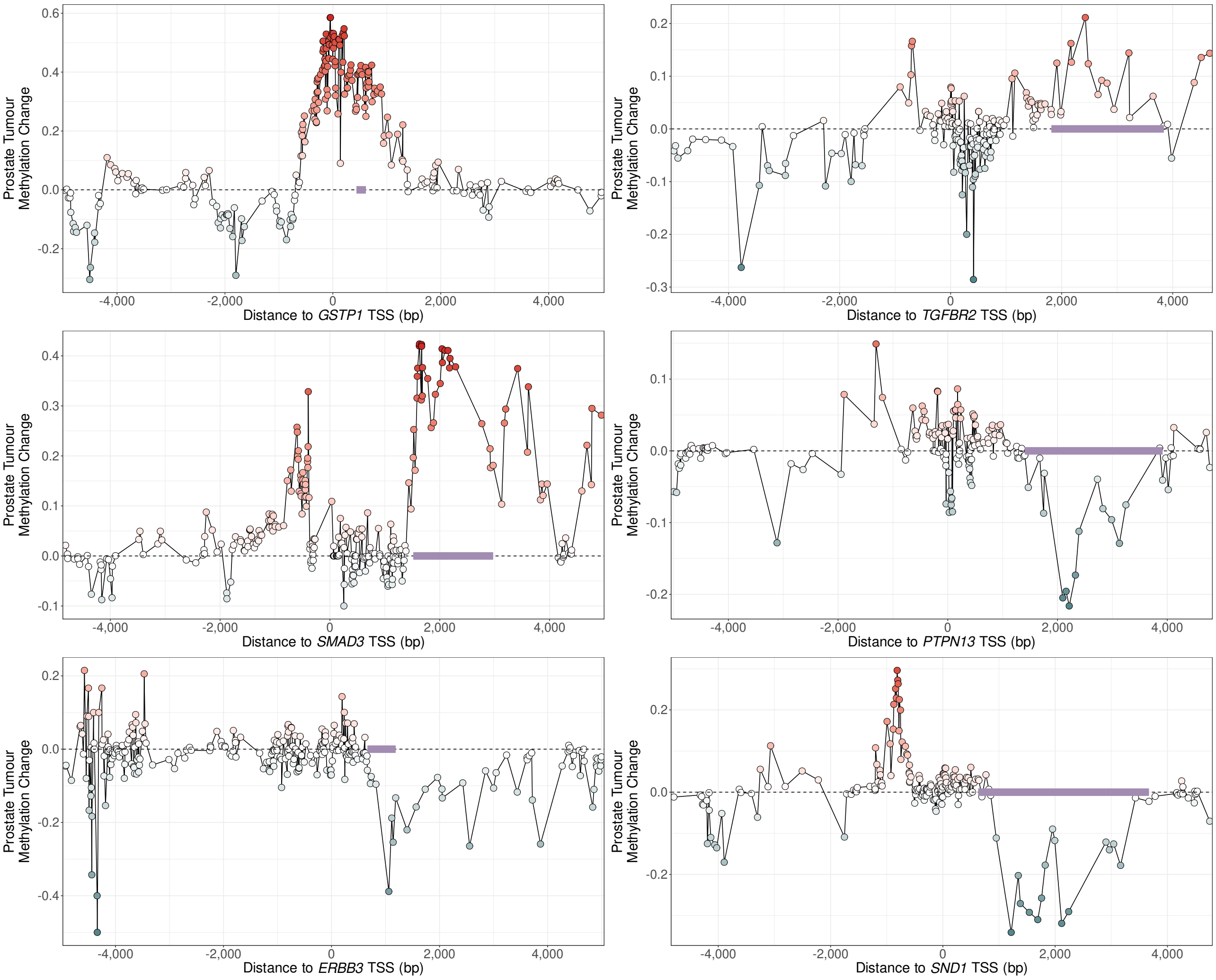

Supplementary Figure 15: Methylation change at negative TMRs associated with *GSTP1*, *TGFBR2*, *SMAD3*, *PTPN13*, *ERBB3* and *SND1* (using the TSS associated with the transcripts ENST00000398606, ENST00000359013, ENST00000327367, ENST00000411767, ENST00000267101 and ENST00000354725) in prostate tumour sample. The x-axes show distance of CpG sites upstream and downstream of the TSS in base pairs and the y-axes show mean methylation change relative to normal prostate samples.

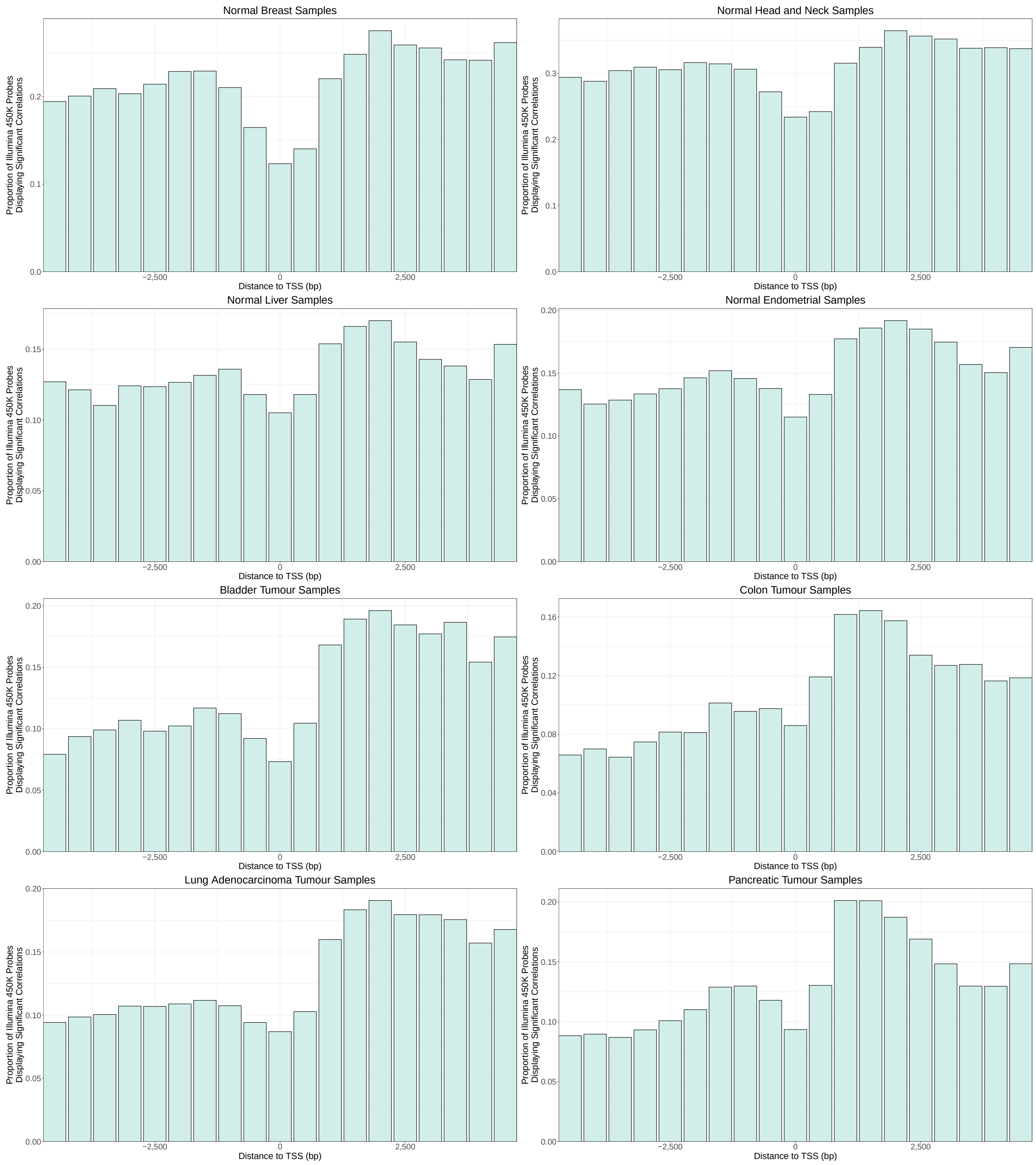

Supplementary Figure 16: The proportion of Illumina 450K probes where DNA methylation was significantly correlated with gene expression in 500 bp bins around TSS in normal and tumour samples from different cancer types from TCGA. The TSS associated with the MANE transcript was used as the TSS for each gene. Significant was defined as an FDR-corrected p-value < 0.05 for the Spearman correlation values. The greatest proportion of significant correlation is generally found downstream of the TSS, with the region at the TSS generally displaying the lowest proportion.

Supplementary Table legends

Supplementary Table 1: All significantly enriched transcriptional regulators for each TMRs group. The enrichment of binding sites among TMR groups was tested by comparing the proportion of CpG sites within TMRs which overlap binding sites with that of all CpGs within the regions +/- 5 kb around the associated TSS using a two-sided chi-squared test. p-values were adjusted using the Benjamini-Hochberg procedure.

Supplementary Table 2: File accession IDs and tissue types for files downloaded from the Roadmap Epigenomics Project.
